# A compartmental model to describe acute medical in-patient flow through a hospital

**DOI:** 10.1016/j.heliyon.2025.e42260

**Published:** 2025-01-24

**Authors:** Tahani Al-Karkhi, Kit Byatt

**Affiliations:** a School of Mathematics, Statistics and Actuarial Science (SMSAS), University of Essex, Wivenhoe Park, Colchester CO4 3SQ, United Kingdom; b Consultant Geriatrician, Independent Medical Practitioner

**Keywords:** Dynamics, Modelling bed usage, Stability analysis, Bifurcation analysis

## Abstract

Managing patient flow in hospitals is a critical part of effective secondary care. Considering a hospital as a multi-compartment system through which patients pass, we will derive relevant equations to allow a description of patient flow through these departments as a series of interconnected dynamic relationships. These relationships are determined by many factors, some known, and many interdependent. We do not need to, and indeed cannot, know all of these factors (as needed for discrete event simulation, or agent-based modelling), but will merely examine the net changes between compartments (i.e. a ‘system dynamics’ approach). Using this approach, we were able to identify two relevant states of equilibrium: the first (trivial) is when the hospital is empty; the second, is when there is activity. We plan to use bed usage data from a UK hospital in an attempt to validate this methodology and then assess its generalizability.

## 1 Introduction

The United Kingdom National Health Service (NHS) is currently under great pressure, not least in relation to hospital bed availability. One of the main factors causing problems in secondary (i.e. hospital) care is that the UK has one of the lowest ratios of hospital beds per capita among Organisation for Economic Co-operation and Development (OECD) countries [3]. As a result, the management of patient flow through hospital has become a major problem. This relative lack of beds, together with increasing demand means that bed occupancy gradually rose from 87*.*7% in quarter 3 of 2010*/*11 to 92% in Q3 of 2019*/*20 (i.e. pre-pandemic) [4].

Health care is becoming increasingly complex, especially in secondary care. Hospital bed numbers often represent the limiting factor in delivering secondary care. Mechanisms to improve the efficiency of their use (and reduce opportunity costs) are therefore important to managers. There have been attempts to model bed usage in departments (micro-level) and across whole health systems (macro-level), however so far, there have been few models developed allowing reliable description and analysis of hospital bed use across a whole hospital (looking at the interactions between parts of a hospital as patients move through) from admission to discharge or death. Patients with serious medical conditions (e.g. COVID-19 [hereafter, Covid]) pass into, through, and out of, hospital. Considering the hospital as a multi-compartment system, we aimed to derive the relevant equations to allow a description of these departments' dynamic relationships. These relationships are determined by many factors, some known, and many interdependent. The primary research question this study addresses is: How can a dynamical systems approach effectively model patient flow through hospital departments to optimize bed usage and improve resource allocation? This work is necessary because existing models of hospital bed usage often focus on specific units or limited scenarios (e.g., emergency departments or COVID-19 patient flows) and do not comprehensively capture the interconnected dynamics of a hospital-wide system.

We do not need to, and indeed cannot, know all of these factors (as needed for discrete event simulation, or agent-based modelling), but will merely look at the net changes between compartments (i.e. a ‘system dynamics’ approach).

**Background** Because of this pressure on hospital beds, hospital managers are always keen to develop systems to maximize their use. To achieve this depends on understanding what sort of patients the system mainly deals with, and how they are moved through the system (patient flow). Fifty years ago, it was identified that using mathematical modelling could help managers to improve bed usage [18]. Various methods, including deterministic and stochastic approaches, multistage models, and algorithmic methodologies, have been used to model bed usage in hospitals. These have all had shortcomings. For example: focusing on planned admissions [26], [5]; being confined to a single (often highly specialised) unit [24], [23], [20], [6], [17]; only part of the medical caseload (and with no definition of the patient groups) [2]; or medium-term planning for a whole hospital [15] or city's healthcare system [22], [7]. Perhaps the most nearly useful model was StratBAM [9], devised for a hospital with fully electronic patient records, in the USA health system—both factors very different from NHS practice. The only systematic review of computer simulation modelling in the field was devoted to examining patient flow within UK emergency departments [20]. Even within that (organisationally) very limited speciality, they found the evidence base to be ‘small and poorly developed’.

The Covid pandemic placed even greater stresses on many hospitals around the world. This generated a number of studies trying to model the impact and help predict bed needs. The focus of these studies varied, but most did not examine all general medical admissions (Covid *and* otherwise) passing through one hospital. A systematic review of hospital surge capacity planning found 690 articles, but identified six models that projected both caseload and hospital capacity requirements over time [14]. None of these examined intra-hospital patient flow patterns. Other papers only considered Covid patients' use of hospital resources (in an effort to optimise use of scarce intensive care resources) [8], national healthcare-wide efficiency of hospital bed management [25], or planning the building of new bed capacity [19].

## 2 General model and description

**Figure 1:**
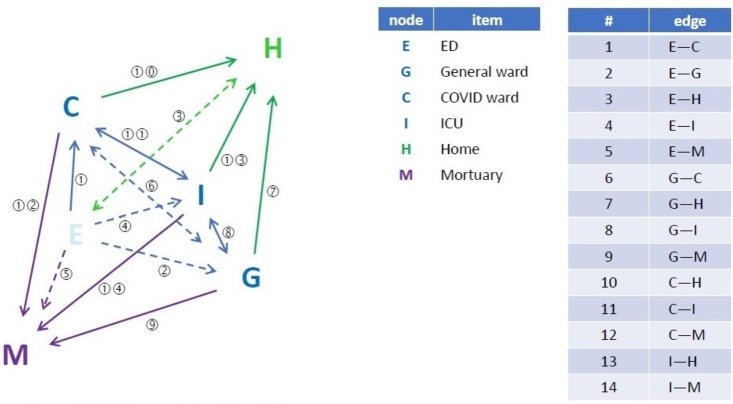
The diagram shows schematically the dynamical equilibrium between the various hospital departments, from admission via the emergency department (E), via different wards (G, C, I), to discharge (H) or death (M).

To answer the question of how waiting in the admissions affects the death rate, we discuss the links between each compartment presented in Fig. 1 as follows:

1. Direct effects There is strong empirical evidence of the importance of the following:
  • The number of beds currently available and used for medical patients, as opposed to patients under other the care of other specialties—e.g. surgery, orthopedics, gynaecology, etc (see above), and
  • The number of patients admitted under the care of the on-call medical team in a 24 hour period. Patients admitted under the care of the medical team will have usually a relatively predictable case mix (heart attacks, strokes, complications of diabetes, infections, etc.). The proportion of each of these conditions, although unpredictable from day to day, and season to season, remains fairly stable over longer periods of time (year to year). It is also well established that the numbers of emergency admissions vary by day of the week, with weekdays having over twice the number of daily admissions than weekends.
2. Indirect effects The low flow in the ED may be a marker of other local cultural or resource challenges. For example, there might be poor trust leadership affecting all other departments. Equally, there may be particular problems with local social services, resulting in delayed discharges of patients medically fit for discharge from all wards, including the ED (i.e. vectors 1–4 in Fig. 1)—as we are seeing in most NHS hospitals, currently.

For example, during the pandemic (up to March 2024) the weekly number of patients admitted to hospital with Covid in England varied from 45 to 3800. The average total number of emergency medical admissions in England for 2020–2021 was about 7,300 in the largest wave of Covid infections to date, the doubling time of hospital admissions in England was 26 days in early March and the halving time was 20 days in April. The number of emergency medical admissions is largely determined by the empty beds available on any given day (the threshold for admission being adjusted to reflect this by the admitting medical team). The proportion of Covid admissions is obviously closely associated with the number of overall cases in the community at any given time. We have made the assumption that knowing the number of patients in the hospital at any given time and the number of available beds, we can estimate the number of cases that will need critical care (i.e., in the ICU) over the subsequent 24 hours. Given that the effective time constant for changes in case mix usually is in the order of months to years, but that for infectious epidemics is in the order of days, and we are modelling changes over a 24 hour period, we can safely assume that non-epidemic case mix is a constant, for the purposes of this model, the various components of the model are presented in Fig. 1. This can thus be presented in Eq. (1) as follows:

(1) $$\frac{dE}{dt}=F_{1}(E,C)=rE\left(1-\frac{E}{K}\right)-\frac{aEC}{1+b_{1}E},\frac{dC}{dt}=F_{2}(E,C,G,I)=\frac{\Gamma_{1}aEC}{1+b_{1}E}-m_{1}C-\frac{\beta GC}{1+b_{2}C}(1+\frac{\zeta I}{1+\epsilon I}),\frac{dG}{dt}=F_{3}(C,G,I)=\frac{\Gamma_{2}\beta IC}{1+b_{2}C}(1+\frac{\zeta I}{1+\epsilon I})-m_{2}G,\frac{dI}{dt}=F_{4}(E,C,I)=\frac{\beta aEC}{1+b_{1}E}-m_{3}I+\omega E.$$

Here $F_{i}$, i=1,2,3,4, is the interaction function of the developed model. The definition of endogenous (dependent) variables is given as follows:

• **E** emergency department population
• **C** specialist ward(s) (here for Covid patients) population
• **G** general medical ward population
• **I** ICU population
• **H** population of patients at home (encapsulated in *ωE* term).
• **m** mortality.

The initial conditions for the model system in Eq (1) are chosen as E(0)≥0, C(0)≥0, G(0)≥0, and I(0)≥0. The model describes the flow of patients into the ED (emergency department). This will depend on the prevalence of the prevailing infection and the flow of patients out of the hospital (discharges and deaths), in a system that is diminished of Covid. The parameter *r* represents the admissions number, *a* is the movement process rate, i.e. the average of time spent on processing the patients from ED to Covid ward (*C*), when there is less number of infections, *a* is half occupation constants, *β* is the linear rate of hospitality (flow) number of patients in the IC unit (*I*), $\Gamma_{i}$ i=1,2, is the patients' flow number between *E* and other departments. *ζ* is the virus changing rate per day, i.e. the infected population in Ed department per day. *ϵ* is a key parameter that we are going to use to reduce the general four compartment model to a special case model, $M_{i},i=1,2,3$ is the death rate and *ω* is the discharge fixed parameter.

In Eq. (1) the first term in this equation of the model we are using the logistic model to describe the hospital's daily performance with *K* as hospital full resources and *rE* refers all medical admissions including all medical condition looked after by a physician, such as patients with heart attacks, strokes, sepsis, segues and complications of diabetes and so forth- the proportion of these individual medical conditions remains relatively constant, is recruitment (admission) rate of the susceptible population and the per capita natural death rate of the population i.e. α=r−d, where the total population is encapsulated in E=C+G+I, where *C* refers to a Covid medical ward, *G* refers to a general medical ward and *I* refers to the IC unit. We can model each compartment of the hospital separately and end with a 7 equations model. However, we simplified the idea into 4-equations model. The second term of the first equation in Eq. (1) is Holling II functional response to describe the movement of patients from the admission department to the Covid medical ward.

We can define the confirmed infected cases in *I*, which is the IC unit, using the Holling II functional response with $\Gamma_{1}$ as patients flow parameter to describe the movement of susceptible cases from Covid department *C* into the intensive care unit *I* as infected population. The death rate *M* used in the second, third and fourth equations of the model in Eq (1) to describe the death happening in different departments, and in practice it depends on the time of recovery process, which can be related to the total infectious individuals seeking treatment, and how many beds are occupied during this event. The third term also represents the increase of death with *β* as a linear death rate *βC* represents the affect of Covid on infected population at the ICU unit, the infected population could also reach its maximum limit by (1+ϵC) factor.

The second and third equations in the model system in Eq (1) represent patients flow from Covid ward *C* to the general medical ward *G* using Holling type II functional response with *a* to describe the time spent transferring patients from one department to other.

The last equation of the model refers to the hospital main source during Covid, the first term describes the movement of patients population between the IC unit and the rehabilitation unit, where *E* refers to the emergency department, *ω* could be defined as the recovery rate or patients leaving the hospital rate and *M* reflects the mortality number per day. There is a minimum of seven compartments in any hospital and patient flow between them is affected by several factors, including:

• Administrative procedures and the quality of service, this is represented by the logistic equation in Eq. (1).
• The clinical workforce establishment (which can be affected in turn by other factors such as sickness, etc), represented in *K* the hospital resources.
• Level of illness or infection in Covid case, defined in the second equation of the model Eq. (1).
• The capacity of each ward (how many patients can be hospitalised in each department + how many beds are available to hospitalised patients), defined in the third and last equation of the model Eq. (1).

## 3 Analysis of the model equilibrium

This section provides a thorough analysis of the original findings and the nature of the equilibrium determined by the system in Eq. (1). We derive analytical expressions to determine the location and stability of the equilibria in the general case, and present results for specific values of K=500 and K=100. In the following section, we will extend this analysis to cover the entire range of 0≤K≤1000

### 3.1 Location of equilibria

The equilibrium points $E(t)=E_{e}$, $C(t)=C_{e}$, $G(t)=G_{e}$ and $I(t)=I_{e}$ of Eq. (1) corresponding to dE/dt=dC/dt=dG/dt=dI/dt=0. From where the steady state can be defined as, in the aspect of the autonomous system $y^{\prime }=f(y)$, as that point, say ϵ>0, where δ>0 such that if, ψ(t) is any solution, of $y^{\prime }=f(y)$ having $\left\|\psi (t)-y_{0}\right\|<\delta$. A solution is then said to exist, that is, $\psi (t)\forall t\geq t_{0}$ and $\left\|\psi (t)-y_{0}\right\|<\epsilon \forall t\geq t_{0}$. Thus stability occurs in two perspectives: stable if there exist a number $\delta_{0}>0|\psi (t)$ is the solution of $y^{\prime }=f(y)$, having $\left\|\psi (t)-y_{0}\right\|<\delta_{0}$ then $\lim_{t\to +\infty }\psi (t)=y_{0}$. From this definition, the system in Eq. (1) possesses five possible points are presented in Table 1, to explain the four different steady state $\left(E_{e},C_{e},G_{e},I_{e}\right)$ obtained in this system.

• The third clinically (irrelevant) equilibrium is
  (2) $$E_{3}=(0,\frac{m_{2}}{\beta \Gamma_{2}-b_{2}m_{2}},\frac{-m_{1}\Gamma_{1}}{\beta \Gamma_{2}-b_{2}m_{2}},0)$$
• The fourth and the general medical ward free equilibrium is given as follows:
  (3) $$E_{4}=(E,C,0,I),E=\frac{1}{a\Gamma_{1}-b_{1}m_{1}}\;\frac{m_{2}}{\beta \Gamma_{2}-b_{2}m_{2}},C=\frac{r\Gamma_{1}\left(ak\Gamma_{1}-kb_{1}m_{1}-m_{1}\right)}{k{\left(a\Gamma_{1}-b_{1}m_{1}\right)}^{2}},G=0,I=\frac{m_{1}\left(a\beta kr\Gamma_{1}-\beta krb_{1}m_{1}+ak\omega \Gamma_{1}-k\omega b_{1}m_{1}-\beta rm_{1}\right)}{km_{3}{\left(a\Gamma_{1}-b_{1}m_{1}\right)}^{2}}.$$
• The fifth and the full dynamical equilibrium is the full dynamical persistence state given by a quartic polynomial as follows;
  (4) $$\sum_{i=0}^{4}A_{i}E_{e}^{4-i}=0,$$
  where $A_{i}$, i=0,..,4 are cascading parameters given in the Appendix A.

**Table 1:** Potential equilibria, both clinically significant and insignificant, of the model system given by Eq. (1).

Equilibrium	Definition	Value in parametrized system	Description
*E*_1_	(*E*_*e*_,*C*_*e*_,*G*_*e*_,*I*_*e*_)	(0,0,0,0)	trivial equilibrium
*E*_2_	(*E*_*e*_,*C*_*e*_,*G*_*e*_,*I*_*e*_)	$\left(K,0,0,\frac{K\omega }{m_{3}}\right)$	hospital resources and the ICU equilibrium
*E*_3_	(*E*_*e*_,*C*_*e*_,*G*_*e*_,*I*_*e*_)	(0,*C*,−*G*,0)	clinically irrelevant point given in Eq. (2)
*E*_4_	(*E*_*e*_,*C*_*e*_,*G*_*e*_,*I*_*e*_)	(*E*,*C*,0,*I*)	general medical ward free equilibrium is given by Eq. (3)
*E*_5_	(*E*_*e*_,*C*_*e*_,*G*_*e*_,*I*_*e*_)	(*E*,*C*,*G*,*I*)	the full dynamical equilibrium is given in Eq. (4)

## 4 Qualitative analysis of the location of the equilibria

The Jacobian of the four-species model is given by

$$J=\left[\begin{array}{cccc} a_{11} & a_{12} & a_{13} & a_{14}\\ a_{21} & a_{22} & a_{23} & a_{24}\\ a_{31} & a_{32} & a_{33} & a_{34}\\ a_{41} & a_{42} & a_{43} & a_{44} \end{array}\right],$$

here, $a_{ij}$, where i,j=1,...,4, represent the partial derivatives of the Jacobian matrix. The stability of the four equilibria is determined by the eigenvalues of this matrix. In the following section, we will explore the behaviour of each equilibrium point.

$a_{1,1}=-\frac{2\;E^{3}r{b_{1}}^{2}-E^{2}(kr{b_{1}}^{2}+4\;rb_{1})-2\;E(krb_{1}+r)-kr+aCk+2\}}{k{\left(Eb_{1}+1\right)}^{2}}$

$a_{1,2}=-\frac{aE}{\left(Eb_{1}+1\right)}$

$a_{1,3}=0$, $a_{1,4}=0$

$a_{2,1}=\frac{\Gamma_{1}aC(1+b_{1}E)-\Gamma_{1}aEC\cdot b_{1}}{{\left(1+b_{1}E\right)}^{2}}$

$a_{2,2}=\frac{\Gamma_{1}aEC}{1+b_{1}E}-m_{1}C-\frac{\beta G(1+b_{2}C)-\beta GC\cdot b_{2}}{{\left(1+b_{2}C\right)}^{2}}\left(1+\frac{\zeta I}{1+\epsilon I}\right)$

$a_{2,3}=-\frac{\beta \;C}{Cb_{2}+1}+\frac{\beta \;C\left(\zeta \right)\;I}{\left(1+I\right)\left(Cb_{2}+\right)}$

$a_{2,4}=\frac{\beta \;GC\zeta }{{\left(1+I\right)}^{2}\left(Cb_{2}+1\right)}$

$a_{3,1}=0$

$a_{3,2}=\frac{\Gamma_{1}aEC}{1+b_{1}E}-m_{1}C-\frac{\beta G(1+b_{2}C)-\beta GC\cdot b_{2}}{{\left(1+b_{2}C\right)}^{2}}\left(1+\frac{\zeta I}{1+\epsilon I}\right)$

$a_{3,3}=-m_{2}$

$a_{3,4}=\frac{\Gamma_{2}\beta C(1+b_{2}C)-\Gamma_{2}\beta IC\cdot b_{2}}{{\left(1+b_{2}C\right)}^{2}}\cdot \frac{\zeta (1+\epsilon I)-\zeta I\cdot \epsilon }{{\left(1+\epsilon I\right)}^{2}}$

$a_{4,1}=\frac{\beta \;aC}{Eb_{1}+1}-\frac{\beta \;aECb_{1}}{{\left(Eb_{1}+\right)}^{2}}+\omega$

$a_{4,2}=\frac{\beta \;aE}{Eb_{1}+1}$

$a_{4,3}=0$

$a_{4,4}=-m_{3}$

**System behaviour near the origin** $E_{1}$

A straightforward calculation shows that the hyperbolic equilibrium or the first trivial equilibrium is a stable fixed point.

(5) $$\lambda_{E_{1}}=(-r,m_{1},m_{2},m_{3})$$

**System behaviour near the hospital resources and the ICU equilibrium** $E_{2}$

The Jacobian matrix of the system Eq (1) around the non feasible point, $E_{2}=(K,0,0,\frac{K\omega }{M_{3}})$ gives the following eigenvalues.

(6) $$\lambda_{1}=-r,\;\lambda_{2}=\frac{ak\Gamma_{1}-kb_{1}m_{1}-m_{1}}{kb_{1}+1},\;\lambda_{3}=-m_{2},\;\lambda_{4}=-m_{3}$$

which is saddle point. The three negative eigenvalues imply stability along three directions in state space, meaning that small perturbations in those directions will tend to return to the equilibrium point over time. The one positive eigenvalue implies instability along one direction in state space, indicating that small perturbations in that direction will grow over time, leading the system away from the equilibrium point. In summary, a saddle point is characterized by a combination of stable and unstable directions.

**System behaviour near the clinically irrelevant point** $E_{3}$ **given in Eq.** (2)

The Jacobian matrix of the system Eq (1) around the non feasible point, for point $E_{3}=(0,C,-G,0)$ gives the following eigenvalues.

(7) $$\lambda_{1}=-\frac{-\beta r\Gamma_{2}+rb_{2}m_{2}+am2}{\beta \Gamma_{2}-b_{2}m_{2}}$$

where $\lambda_{2,3}$ is given in Eq (8) as follows:

(8) $$\lambda_{2,3}=\pm \frac{1}{2}\frac{-m_{1}m_{2}b_{2}+\sqrt{4\beta^{2}\Gamma_{2}^{2}m_{1}m_{2}-4\beta \Gamma_{2}b_{2}m_{1}m_{2}^{2}+b_{2}^{2}m_{1}^{2}m_{2}^{2}}}{\beta \Gamma_{2}}$$

(9) $$\lambda_{4}=-m_{3}$$

The equilibrium is a saddle-focus point, exhibiting a combination of stability and instability along with oscillatory behaviour. The real eigenvalues govern the stability aspects, while the complex conjugate eigenvalues contribute to the oscillatory dynamics.

**System behaviour near general medical ward free equilibrium** $E_{4}$ **is given by Eq.** (3)

The Jacobian matrix of the system Eq. (1) for the fourth equilibrium with no population in the general medical ward G=0.

(10) $$\lambda_{1}=\frac{-\alpha }{\beta }$$

where *α* and *β* are two cascading parameters given in Eq. (23) and Eq. (24) in the Appendix C.

(11) $$\lambda_{2,3}=\pm \frac{\left(a\Gamma_{1}-b_{1}m_{1}\right)}{2ak\Gamma_{1}}\;\sqrt{a^{2}k\Gamma^{2}m_{1}-a^{2}k\Gamma_{2}^{2}m_{1}-akr\Gamma_{1}b_{1}m_{1}-ak\Gamma_{1}b_{1}m_{1}^{2}+ak\Gamma_{1}b_{1}m_{1}^{2}}+krb_{1}^{2}m_{1}^{2}+rm_{1}a\Gamma_{1}+rb_{1}m_{1}^{2}-4a^{4}k^{2}r\Gamma_{1}^{4}m_{1}+a^{4}k^{2}\Gamma_{1}^{4}m_{1}^{2}-(2a^{4}k^{2}\Gamma_{1}^{4}m_{1}^{2}+a^{4}k^{2}\Gamma_{1}^{4}m_{1}^{2}+2a^{3}k^{2}r\Gamma_{1}^{3}b_{1}m_{1}^{2}+10a^{3}k^{2}r\Gamma_{1}^{2}\Gamma_{1}b_{1}m_{1}^{2}-2a^{3}k^{2}\Gamma_{1}^{3}b_{1}m_{1}^{3}+4a^{3}k^{2}\Gamma_{1}^{3}b_{1}m_{1}^{3}-2a^{3}k^{2}\Gamma_{1}^{3})b_{1}m_{1}^{3}\;a^{2}k^{2}r^{2}\Gamma_{1}^{2}b_{1}^{2}m_{1}^{2}-4a^{2}k^{2}r\Gamma_{1}^{2}b_{1}^{2}m_{1}^{3}-(8a^{2}k^{2}r\Gamma_{1}^{2})b_{1}^{2}m_{1}^{3}+a^{2}k^{2}\Gamma_{1}^{2}b_{1}^{2}m_{1}^{4}-(2a^{2}k^{2}\Gamma_{1}^{2}b_{1}^{2}m_{1}^{4}+a^{2}k^{2}\Gamma_{1}^{2}b_{1}^{2}m_{1}^{4}-2ak^{2}r^{2}\Gamma_{1}b_{1}^{3}m_{1}^{3}+2ak^{2}r\Gamma_{1}b_{1}^{3}m_{1}^{4}+2ak^{2}r\Gamma_{1}b_{1}^{3}m_{1}^{4}+k^{2}r^{2}b_{1}^{4}m_{1}^{4}-2a^{3}kr\Gamma_{1}^{3}m_{1}^{2}+6a^{3}kr\Gamma_{1}^{2}\Gamma_{1})m_{1}^{2}-2a^{2}kr^{2}\Gamma_{1}^{2}b_{1}m_{1}^{2}-(8a^{2}kr\Gamma_{1}^{2}b_{1}m_{1}^{3}+2akr\Gamma_{1}b_{1}^{2}m_{1}^{4}+2akr\Gamma_{1}b_{1}m_{1}^{4}+2kr^{2}b_{1}^{3}m_{1}+a^{2}r^{2}\Gamma_{1}^{2}m_{1}^{2}+2ar^{2}\Gamma_{1}b_{1}m_{1}+r^{2}b_{1}m_{1}.$$

(12) $$\lambda_{4}=-m_{3}$$

The eigenvalues are unstable saddle focus because we obtained four eigenvalues with opposite signs. We could also identify this point as a saddle-node or bifurcation point. The presence of two negative real eigenvalues implies stability along two directions in state space. Small perturbations in those directions will tend to return to the equilibrium point over time. Complex conjugate eigenvalues with different signs (onepositive,onenegative) indicate a behaviour akin to a focus. This suggests that trajectories near the equilibrium point exhibit spiralling behaviour.

**System behaviour near the full dynamical equilibrium** $E_{5}$ **is given in Eq.** (4)

The Jacobian matrix $J_{4}={\left(a_{ij}\right)}_{4\times 4}$ is given in section 4 Let $\lambda_{i}$, i=1,2,3,4 be the roots of the characteristic polynomially of $J_{4}$ which is given by:

(13) $$\sum_{i=0}^{4}A_{i}\lambda^{4-i}=0,$$

where $A_{i}$ are cascading parameters and $A_{0}=1$;

(14) $$A_{1}=-a_{1,1}-a_{2,2}-a_{3,3}-a_{4,4}.$$

(15) $$A_{2}=a_{1,1}a_{2,2}+a_{1,1}a_{3,3}+a_{1,1}a_{4,4}-a_{1,2}a_{2,1}+a_{2,2}a_{3,3}+a_{2,2}a_{4,4}-a_{2,3}a_{3,2}-a_{2,4}a_{4,2}+a_{3,3}a_{4,4}.$$

(16) $$A_{3}=-a_{1,1}a_{2,2}a_{3,3}-a_{1,1}a_{2,2}a_{4,4}+a_{1,1}a_{2,3}a_{3,2}+a_{1,1}a_{2,4}a_{4,2}-a_{1,1}a_{3,3}a_{4,4}+a_{1,2}a_{2,1}a_{3,3}+a_{1,2}a_{2,1}a_{4,4}-a_{1,2}a_{2,4}a_{4,1}-a_{2,2}a_{3,3}a_{4,4}+a_{2,3}a_{3,2}a_{4,4}-a_{2,3}a_{3,4}a_{4,2}+a_{2,4}a_{3,3}a_{4,2}.$$

(17) $$A_{4}=a_{1,1}a_{2,2}a_{3,3}a_{4,4}-a_{1,1}a_{2,3}a_{3,2}a_{4,4}+a_{1,1}a_{2,3}a_{3,4}a_{4,2}-a_{1,1}a_{2,4}a_{3,3}a_{4,2}-a_{1,2}a_{2,1}a_{3,3}a_{4,4}-a_{1,2}a_{2,3}a_{3,4}a_{4,1}+a_{1,2}a_{2,4}a_{3,3}a_{4,1}.$$

According to the Routh-Hurwitz criterion, the condition for all eigenvalues of the Jacobian matrix to have negative real parts is equivalent to the determinant of all the Hurwitz matrices being positive. This implies that any equilibrium *E* is locally asymptotically stable if and only if $A_{1}>0$, $A_{3}>0$, $A_{1}A_{2}>A_{3}$, and $A_{3}>\sqrt{A_{1}(A_{1}A_{4}-A_{2}A_{3})}$, or $A_{1}A_{2}A_{3}>A_{3}^{2}+A_{1}^{2}A_{4}$. Clearly, we have $A_{1}<0$ and $A_{3}<0$, and based on the Jacobian matrix elements, when $a_{1,2}<0$, $a_{2,1}>0$, $a_{2,3}<0$, $a_{3,2}>0$, $a_{3,3}<0$, and $a_{4,4}<0$, it follows that $A_{1}A_{2}A_{3}>A_{3}^{2}+A_{1}^{2}A_{4}$. Therefore, using the Routh-Hurwitz criteria, we derive the necessary and sufficient conditions for the positive equilibrium to be locally asymptotically stable. To facilitate this, we introduce the following notation.

$a_{1,1}>0$ i.e. if $\alpha >\frac{aG_{e}}{\left(1+b_{1}E_{e}\right)}+\frac{aE_{e}I_{e}b_{1}}{{\left(1+b_{1}E_{e}\right)}^{2}}(\frac{k}{\left(1-2E_{e}\right)})$ and $a_{1,2}<0$ i.e. if $\frac{-aE_{e}}{\left(1+b_{1}E_{e}\right)}<0$ and

$a_{2,1}>0$ i.e. $b_{1}<\frac{E_{e}}{\left(1+b_{1}E_{e}\right)}$ and $a_{2,3}<0$ if $\zeta <\frac{C_{e}-1}{C_{e}}$ and $a_{4,4}<0$. Hence $E_{4}$ is unstable Equilibria. This suggests that trajectories near the equilibrium point exhibit spiralling behaviour. The stability of this spiralling behaviour depends on the sign of the real part of the complex eigenvalues, which indicates one positive real part in the complex eigenvalues roots. If one complex eigenvalue has a positive real part and the other has a negative real part, the equilibrium is considered a “saddle-point focus.”

The system analysis produced five equilibriums. The full dynamical equilibrium is the most relevant as all populations are there and with positive signs and the solution exists at $E_{4}$, which means, the ‘nowcasting’ and how variations in bed usage in the different departments will affect the other parts of the system, both upstream and downstream (‘forecasting’). The upstream is when the bed of the discharge or mortuary while downstream means, beds in active departments, they are also called level 3 beds, more details are provided in Appendix B.

We investigated the equilibria of the system, denoted as $\left(E_{1},E_{2},E_{3},E_{4},E_{5}\right)$, and examined their feasibility under biologically relevant conditions. To ensure the model produces biologically meaningful (non-negative) solutions, we define all initial conditions:

E(0),C(0),G(0),I(0)

and parameters:

$$r,K,a,\beta ,\zeta ,m_{1},m_{2},m_{3},\omega$$

as non-negative, reflecting their real-world interpretations. The terms in the equations, such as:

$$\frac{aEC}{1+b_{1}E},$$

are constructed to remain non-negative under these conditions. Furthermore, the death rates:

$$m_{1}C,\;m_{2}G,\;m_{3}I,$$

are proportional to the populations and cannot drive the variables below zero. The eigenvalues from the stability analysis confirm the biologically meaningful equilibrium ($E_{5}$) is locally stable, and our numerical simulations consistently show all state variables remain non-negative throughout the system's evolution. This guarantees the model adheres to the requirement for biologically meaningful solutions under the given parameter conditions.

## 5 Parameter values investigation

A major reason for modelling the dynamics of a population is to understand its principle controlling features and so be able to predict the likely pattern of development consequent upon a change of environmental parameters [13]. In the hospital's bed model of Eq. (1) we assumed that the parameters within the elementary analysis are the real number of population in the daily entry basis to hospital. Due to the high bed occupancy in UK hospitals, the limiting factor for the number of admissions in any 24 hour period is determined by the beds becoming available that day (i.e. the number of deaths and discharges). Generally, seriously ill patients are assessed in the emergency department and then a decision is made to admit to a medical ward or to send home. If admitted, they will either be sent to a general medical ward *G* or a Covid ward *C*, through out the median number of ward transfers from the ED to medical wards, which are $\Gamma_{1}$ and $\Gamma_{2}$. There is a target that any admissions from the ED should occur within 4 hours; this ‘time to admit’ is *a*. Patients who deteriorate in medical wards and need more intensive treatment are transferred to ICU. The median number of such transfers each day over the preceding week *ζ* reflects this component of activity. The median number of medical patients who die in unit time—in this case one day—in any given ward reflects the case mix on that ward, can be known from hospital data and is represented here by $m_{1}$, $m_{2}$ and $m_{3}$ for the general ward, Covid ward and ICU, respectively. As can be seen, the total number of beds thus is the sum of the number of discharges and deaths in a given period *ω*. Because of the physical limit of beds and staff on any given day, the threshold for admission can change to prevent more patients being admitted than the available resources. As the model in Eq. (1) describes the flow of patients into the hospital via ED (i.e. admission numbers). This number is normally determined by: flow of patients out of hospital (discharges and deaths) in a steady state system in the absence of Covid. The following is a list of Exogenous (independent) parameter values definition

## 6 Numerical simulation results

### 6.1 Time series and phase portraits

This section aims to validate the analytical findings by incorporating experimental parameter values provided by a clinician consultant with hospital experience. For this analysis, we utilized the parameter values listed in Table 2, which outlines the 14 parameters used to examine the model described in Eq. (1). These values also assisted in setting the initial conditions for conducting the numerical analysis. Another key objective of this section is to confirm the analytical results (summarized in Table 1) through numerical methods. The simulations highlight several significant features of the system from a practical perspective.

**Table 2:** Definitions of parameters and their associated units utilised in the presented model in Eq. (1).

Parameters	Definition	Value	Units
*r*	The number of admissions in the past 24 hours	5	no unit
*K*	Hospital resources	100 − 200	no unit
*a*	The movement process rate, i.e the average of time spent on processing the patients from ED to hospital ward, when there is little *Covid*	4	hours
*ζ*	The linear rate of hospitality (flow) number of patients in the ICU	0.2	no unit
*ϵ*	A key parameter that we are going to use to reduce the general four compartment model to a special case model	0 or 1	no unit
Γ_1,2_	The patients flow number between ED and other departments	4 − 5	hours
*β*	The number of available hospital beds (is the discharge fixed parameter)	5	no unit
*b*_1,2_	(bed time handling)	1.5 − 2	days
*m*_1,2,3_	Death rate	1.5	no unit
*ω*	Patients discharge rate from the hospital	3.5	no unit

**Figure 2:**
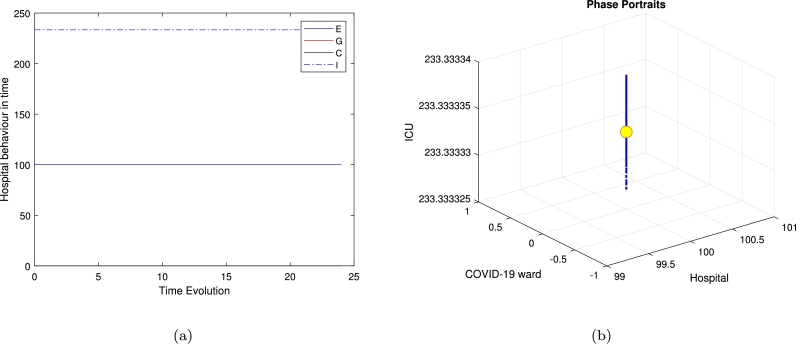
Time series and phase portraits near the equilibrium point (hospital resources and the ICU equilibrium *E*_2_), which is given by (*H*_*e*_,*W*_*e*_,*G*_*e*_,*C*_*e*_)=(100,0,0,233.3333333) with *ζ* = 0.2 and all other parameters fixed as in Table 2. It is readily seen that the trajectories are attracted onto a stable limit cycle.

Fig. 2 exhibits the local stability of the model around the second equilibrium $E_{2}$, while there is no population in the Covid ward and in the general medical ward, the system exhibits a stable equilibrium and all trajectories are moving toward the equilibrium point. The oscillator behaviour is influenced by the second positive root of the quartic polynomial, shaping the stability of the clinically relevant equilibrium $E_{4}$. In the 24-hour case, oscillations reflect short-term periodic dynamics, while in the 500-hour case, they persist over extended durations, indicating long-term regulatory effects. Similarly, Fig. 6 highlights oscillatory trajectories, emphasizing their dependence on system parameters and initial conditions.

Figure 4, Figure 6 illustrate the system's behaviour over continuous time for the special case model, along with its trajectory in *HWC* space based on three distinct initial conditions: $\left(H_{e},W_{e},G_{e},C_{e})=(0.2597,1.2983,0,0.49379\right)$, (0.15810,1.5440,7.6186,3.0031), and (99.9214,0.14818,4.5320,1565.697), with ζ=0.2 and bed numbers (along with all other parameters) fixed at their default values. In Fig. 4(b), the trajectory is shown to converge onto a limit cycle with an approximate period of 500 hours. This trajectory reveals significant large amplitude oscillations in *H*. These findings demonstrate the system's inherent instability.

These results have tended to focus on bed usage within departments (micro-activity), but not activity across healthcare systems (macroactivity). We are looking at the use of beds between departments within a hospital trust (what we call ‘meso-activity’). The results in Figure 4, Figure 5, Figure 6 show the hospital activities in 24 hours, where almost all departments are under control and the patients' movement is stable. As soon as we increase the time's loop to 500 hours, which is approximately 20 days we can notice the periodic behaviour of the system and the flow of the trajectories to create a limit cycle around the three feasible clinically relevant equilibrium. Normally in the UK, emergency cases, including Covid patients, arrive in hospital either after having called for help and been assessed at home by a clinician (usually a paramedic; occasionally a GP), or after having presented at the emergency department (ED) of their local hospital. They are assessed as to how serious and urgent their condition is a process called triage, based on the military practice of rapidly dividing casualties into three groups: those who will survive without intervention; those who will die anyway; and those who will die without intervention but are likely to survive with it.

### 6.2 One parameter bifurcation behaviour

We have used numerical simulations to analyse the proposed model in Eq. (1). Fig. 7 represents the local stability diagram around $E_{4}$ with the parameter values given in Table 2. It is readily seen that in Fig. 7(a) indicates that if in the clinically relevant equilibrium $E_{4}<K$ then the hospital population will increase and if $E_{4}>K$ this means there will be a deficiency in hospital resources. Especially, if $E_{4}$ is a function of *ζ* i.e. it depends tremendously on the number of beds in hospital. Also, if $E_{4}=K$ then the population will remain constant.

These graphs illustrate E−C−G−I clinical model: For a specific choice of parameters, in each cycle, the prey population is increased to extremely high numbers, yet recovers (while the emergency population remains sizeable at the highest number of admitted population density). In real-life situations, however, chance fluctuations of the discrete numbers of individual structure and life-cycle of admitted people might cause the population in *E* to actually increase more than usual due to the cases admitted because of Covid as well as other health conditions. For a specific choice of parameters, the patients population is increases to extremely high numbers, yet recovers (while the *G* and *C* populations remain size able at the highest patients density). In real-life situations, however, chance fluctuations of the discrete numbers of individual structure and life-cycle of virus might cause the patients to actually decease.

## 7 Heat map for the hospital dynamics

To study the effect of altering the medical resources *k* of the hospital on the population dynamics by varying the value of *k* and *ζ* number of beds in the hospital in 24 hours. We presented a heat-map of a hospital in Fig. 7 typically occurs when the system dynamics of Eq. (1) are cyclic, which means when the density of a patients population increases in response to a greater number of infected patients i.e. when there is a surge in the virus and limited resources the death ratio will increase consequently as a response to this mechanism. The patients population density in the general medical ward will remain constant over short timescales but it does support our assumption that the surge in the virus may respond to the limited resources available in the hospital. The heat map of Fig. 8 effect also implies unreasonable responses to changes in patient population in each ward and other parameters affecting population stability such as the hospital resources *k* and *ζ*. It is also defined as response to altered mortality. The different levels of virus effect determine the stabilization of patients population densities in each ward. From the heat map images, an equilibrium is highlighted that indicates an unstable state to the left of the curve of Fig. 8(a) which represent the population densities of *E* ward and the *C* ward in Fig. 8(b) are high and this is reflected in the red colour represents the maximum densities of the equilibria. From this, the results are an increase of both ward's populations. The heat-map in each of Fig. 8(a), Fig. 8(b), Fig. 8(c) and Fig. 8(d) indicates that after crossing the saddle-node curve there is then an unstable equilibrium density. This is even though when there is an increase in the patient flow it will generate pressure on the hospital's medical resources and consequently affect the patient population density in each ward.

## 8 Results and discussion

Several researchers have explored various models of the Holling type II functional response since the groundbreaking contributions of Freedman [16], [10]. These studies aimed to enhance understanding of the dynamics and behaviours of such systems. More recent work, such as Freedman [11], investigates the existence of periodic oscillations and examines the impact of the functional response on these behaviours [1]. In this study, we focus on analyzing the trajectory directions by solving the model represented by Eq. (1). To summarise the analytical findings, we used Table 1 to obtain some numerical results generated using ODE45 in MATLAB. The model in Eq. (1) exhibits four hyperbolic, clinically feasible equilibria and one non-feasible equilibrium $E_{3}$. The first one is $E_{1}$ where all hospital populations are extinct, and this is always stable. The second equilibrium is $E_{2}$, where the ED department is at its highest population density, while both Covid and the general medical ward are empty or with an extinct population and the population in the ICU is limited; this is also stable. The third equilibrium is $E_{3}$ is clinically irrelevant as one of the hospital departments has a negative population. The fourth equilibrium is the clinically relevant, $E_{4}$, which is a quartic polynomial with four real roots two of which are negative roots and three positive where all populations continue to survive. The three positive roots exhibit three different behaviours. The equilibrium $E_{4}$ is a saddle-focus point, exhibiting a combination of stability and instability along with oscillatory behaviour. The real eigenvalues govern the stability aspects, while the complex conjugate eigenvalues contribute to the oscillatory dynamics. Fig. 4 shows the behaviour for the general medical ward free equilibrium in Fig. 4(a) and Fig. 4(b). Fig. 4(c) and Fig. 4(d) of the first positive root of the quartic polynomial, the clinically relevant equilibrium $E_{4}$ in duration of 24 hours and 500 hours cases, which is represented in Eq. (13) and all of the other parameters are fixed as in Table 2.

The parameter set in Table 2 enables a diverse range of system behaviour to emerge within a relatively narrow *ζ* parameter space. The system's stability can be classified into four distinct categories based on hospital population dynamics or limit-cycle patterns [12]. An ‘unstable spiral’ is observed to diverge from the initial conditions, resulting in unbounded population oscillations. We identify a region of such oscillatory behaviour in the four-species model and demonstrate its persistence across the parameter values listed in Table 2. These findings align with those reported in [10].

The oscillatory nature of the hospital model primarily arises due to the complexities involved in modelling the ICU bed functional response (*C*), which plays a critical role in the system's dynamics and is clinically significant [10]. According to [21], using a simple reaction-diffusion model can effectively capture the intricate dynamics, including oscillatory behaviours, by emphasizing trophic interactions.

**Figure 3:**
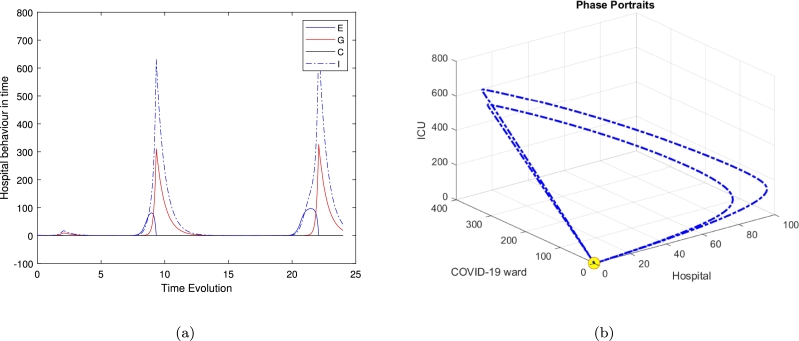
Time series and phase portraits near the equilibrium point (General ward free equilibrium *E*_3_), which is given by (*H*_*e*_,*W*_*e*_,*G*_*e*_,*C*_*e*_)=(0.02597402597,1.298363974,0,0.4934840052) with *ζ* = 0.2 and all other parameters fixed as in Table 2. It is readily seen that the trajectories are attracted onto unstable limit cycle, along with oscillatory behaviour.

Interestingly, the limit-cycle behaviour observed for the linear mortality function of the COVID ward (*W*), the general medical ward (*G*), and the ICU (*C*) may not manifest if a quadratic mortality function is applied to *G* and *C*. The results presented—calculated numerically using ODE45 for various equilibria and illustrated in Fig. 2 for the time series in Fig. 2(a) and phase portraits 2(b), near the equilibrium point (hospital resources and the ICU equilibrium $E_{2}$), with ζ=0.2 and all other parameters fixed as in 2, show all trajectories are attracted onto a stable limit cycle. Fig. 3, is the system behaviour near the equilibrium point (General ward free equilibrium $E_{3}$), with ζ=0.2 and all other parameters fixed as in 2. It shows the trajectories are attracted onto unstable limit cycle via the time series Fig. 3(a) and phase portrait in Fig. 3(b), along with oscillatory behaviour.

**Figure 4:**
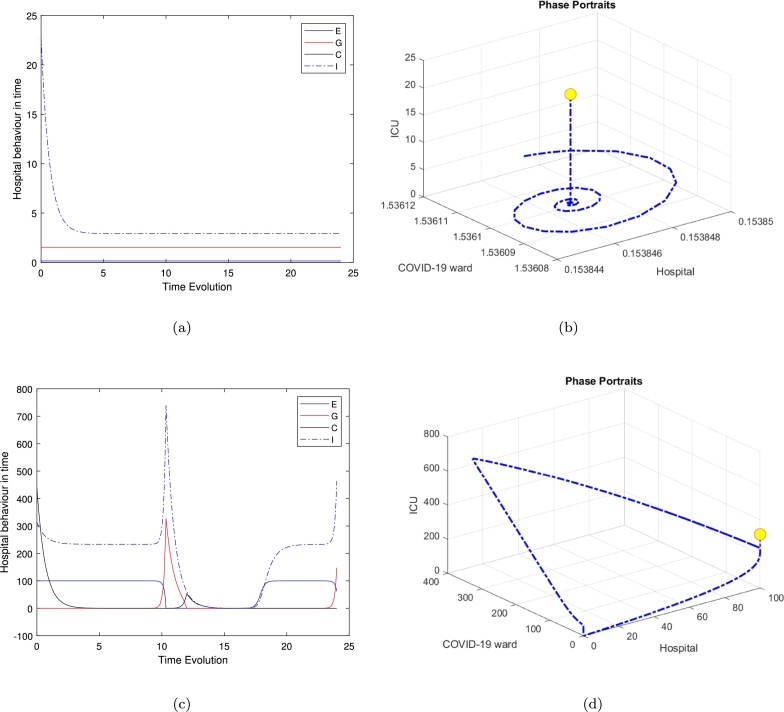
Time series and phase-space trajectories around the proposed initial condition for the general medical ward free equilibrium in Fig. 4(a) and 4(b). Fig. 4(c) and Fig. 4(d) the first positive root of the quartic polynomial, the clinically relevant equilibrium *E*_4_ in duration of 24 hours and 500 hours cases, which is represented in Eq (13) and all of the other parameters are fixed as in Table 2.

**Figure 5:**
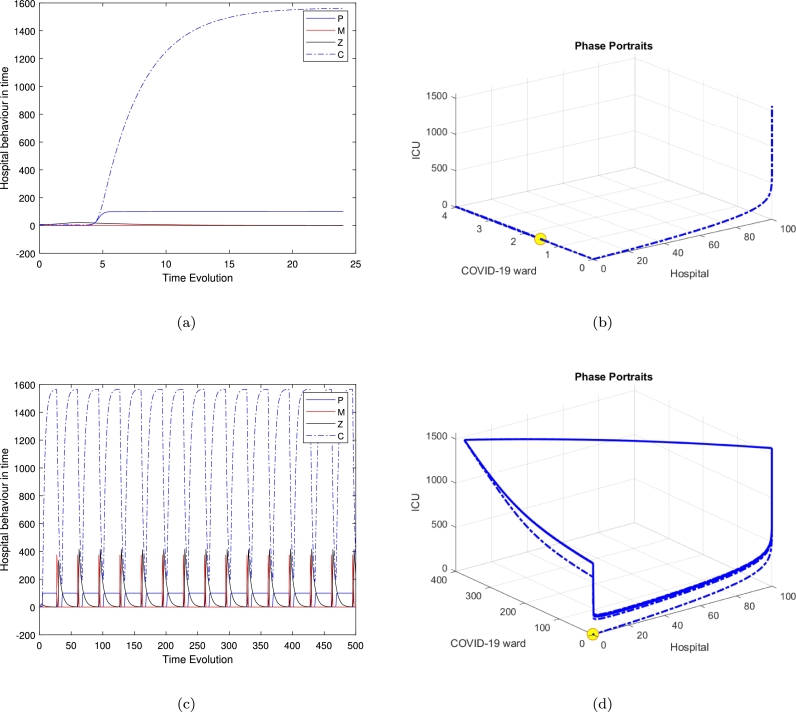
Time series and phase-space trajectories around the proposed initial condition for the second positive root of the quartic polynomial, the clinically relevant equilibrium *E*_4_ in duration of 24 hours and 500 hours cases, which is represented in Eq (13) and all of the other parameters are fixed as in Table 2.

**Figure 6:**
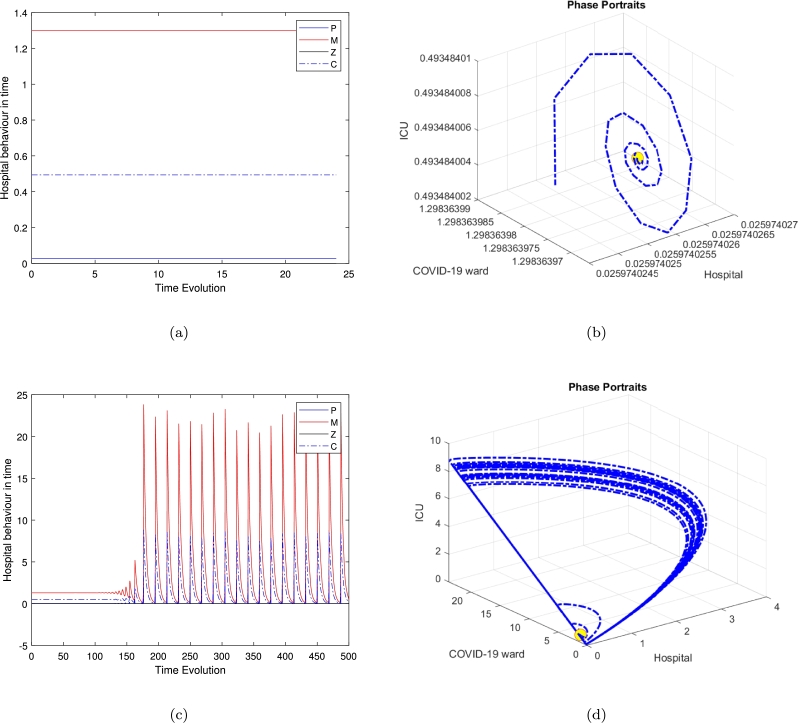
Time series and phase-space trajectories around the proposed initial condition for the third positive root of the quartic polynomial, the clinically relevant equilibrium *E*_4_ in duration of 24 hours and 500 hours cases, which is represented in Eq (13) and all of the other parameters are fixed as in Table 2.

In Figs. 4(a), 4(b), 4(c) and 4(d), highlight these dynamics, we observed that the clinically relevant equilibrium is unstable when ζ=2.3. While Figs. 5(a), 5(b), 5(c), 5(d), which refer to the second positive root of the quartic polynomial, the clinically relevant equilibrium $E_{4}$ in duration of 24 hours and 500 hours cases, and similarly in Fig. 6.

The model in Eq. (1) displays a region of instability near the Hopf bifurcations when K=20, r=4.455, ζ=2.3 and ω=1.5. However, this model does not exhibit behaviour in accordance with the enrichment paradox. While increasing the hospital's main resources *K* does take the system through a region of unstable states, the presence of a higher population in both *W* and *C* causes the system to be unstable for larger values of *K*. This captures detailed information on interactions among the three departments.

## 9 Conclusion

**Figure 7:**
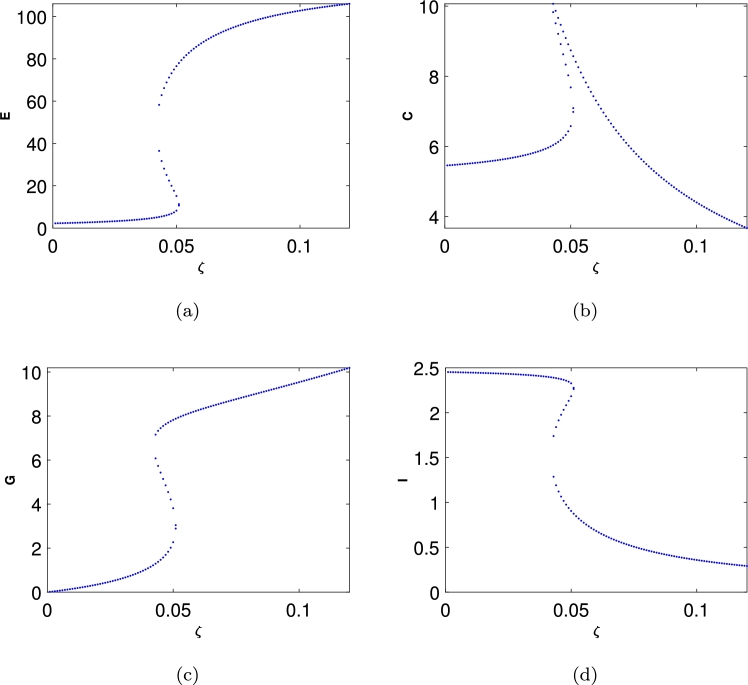
The clinically relevant equilibrium point w.r.t. *ζ* as a bed number parameter in a hospital.

The primary goal of this analysis is to gain deeper insights into the hospital dynamics described by the model in Eq. (1). This study confirms the existence of periodic oscillations in solutions and evaluates how the virus response influences hospital operations. It highlights the importance of understanding the impact of COVID-19 spread within the structure of hospital wards. In this investigation, the trajectory directions were analyzed by solving the model in Eq. (1). To summarize the analytical results, Table 1 was used to present numerical findings obtained through MATLAB's ODE45 solver. The model in Eq. (1) reveals four hyperbolic, clinically feasible equilibria and one non-viable equilibrium, $E_{3}$. The first equilibrium, $E_{1}$, where all populations are extinct (i.e. the hospital is empty), is always unstable. This study aims to enhance understanding of hospital dynamics during the COVID-19 pandemic, particularly under the constraints of limited bed availability. It also examines the stabilization of hospital operations by focusing on the bed usage in each ward, particularly the ICU. The second equilibrium is $E_{2}$ where the patients in *E* are at the highest population while the patients in *C* and the *G* are no longer present, and the spread of Covid is constrained, making it unstable, as described in Section 4. The third one is $E_{3}$ where the patients and Covid spread persist while the hospital resources are extinct. The fourth equilibrium is the coexistence $E_{4}$ where all wards populations exist as explained in sec. 3. The parameter values in Table 2 facilitate observing diverse behaviour within a relatively narrow *ζ* parameter space. The system's stability can be classified into four distinct categories, based on the E−C−G−I population dynamics or the limit-cycle graph behaviour [12]. An unstable spiral emerges from the initial condition, leading to unbounded population oscillations. We demonstrate the presence of this oscillatory region in the four-species model and show how it persists with the parameter values specified in Table 2. The oscillatory behaviour in the E−C−G−I model is the challenge of modelling using Holling type II functional response, as it is clinically intricate and possibly impactful on the system. We chose the Holling Type II functional response to represent patient transfers between departments because it effectively captures the saturation effect observed in hospital systems, where the rate of patient transfer slows as departmental capacity approaches its limit. The observed limit-cycle behaviour with respect to linear mortality for patients may not appear if a quadratic mortality function is applied to each ward [1]. The numerical results shown in Fig. 7, computed using ODE45 for different equilibria, reveal that the coexistence equilibrium becomes unstable when ζ=0.001. In this case, a neutral centre corresponds to a closed loop, where population oscillations persist without damping. A stable spiral gradually moves toward a stable state, with oscillations in the population being dampened until all populations stabilize. We intend to validate these findings using real-world hospital patient flow data.

**Figure 8:**
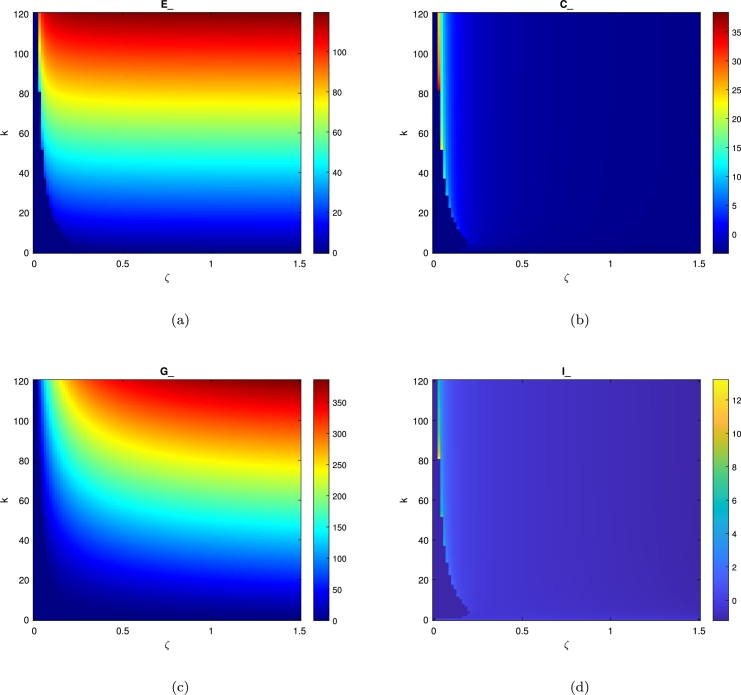
A heat map to represent the hospital dynamics and stability during 24 hours, which indicate the system maximum equilibrium (*E*_*e*_,*C*_*e*_,*G*_*e*_,*I*_*e*_) for the parameters for different values of *K* and *ζ*. Fig. 8(a) shows the emergency department *E* heat map which reflects the persistence of a high level of population in this department represented by the red colour. Fig. 8(d) represents the low population in the *ICU* unit due to the limited beds number.

## CRediT authorship contribution statement

**Tahani Al-Karkhi:** Validation, Methodology, Formal analysis. **Kit Byatt:** Writing – original draft, Project administration.

## Declaration of Competing Interest

The authors declare that they have no known competing financial interests or personal relationships that could have appeared to influence the work reported in this paper.

## Data Availability

No new data was generated for the research described in the article.

## Appendix A

• The coefficient of the full dynamic equilibrium polynomial given in Eq. (4):
  (18) $$A_{1}=\left(\alpha^{2}b^{2}d_{3}+\Gamma_{1}^{2}+\alpha^{2}b^{2}d_{C}\Gamma_{1}^{2}\right)$$
  (19) $$A_{2}=\left(-2K\alpha^{2}b^{2}d_{3}+\Gamma_{1}^{2}-2K\alpha^{2}b^{2}d_{C}\Gamma_{1}^{2}+\alpha^{2}bd_{3}+\Gamma_{1}^{2}+\alpha^{2}bd_{C}\Gamma_{1}^{2}\right)$$
  (20) $$A_{3}=K^{2}r^{2}b^{2}d_{3}+\Gamma_{1}^{2}+K^{2}r^{2}b^{2}d_{3}\Gamma_{1}^{2}-2Kr^{2}bd_{3}+\Gamma_{1}^{2}-2Kr^{2}bd_{3}C\Gamma_{1}^{2}+Krb^{2}d_{1}+d_{3}\Gamma_{1}+Kr+b^{2}d_{1}-d_{3}C\Gamma_{1}+Krb^{2}d_{3}+d_{3}C\Gamma_{1}+Krb^{2}d_{3}d_{2}CC\Gamma_{1}+Karbd_{2}+Karbd_{2}-Kard_{3}\Gamma_{1}^{2}-Kard_{3}C\Gamma_{1}^{2}$$
  (21) $$A_{4}=K^{2}r^{2}bd_{3}\Gamma_{1}^{2}+K^{2}r^{2}bd_{3}C\Gamma_{1}^{2}-K^{2}rb^{2}d_{1}d_{3}+\Gamma_{1}-K^{2}r\;b^{2}d_{1}d_{3}C\Gamma_{1}-K^{2}r\;b^{2}d_{3}d_{2}C\Gamma_{1}-K^{2}r+b^{2}d_{3}Cd_{2}C\Gamma_{1}-d_{2}braK^{2}-K^{2}arbd_{2}G+K^{2}ard_{3}\Gamma_{1}^{2}+K^{2}ard_{3}C\Gamma_{1}^{2}+Krbd_{1}d_{3}\Gamma_{1}+Krbd_{1}d_{2}C\Gamma_{1}+Krbd_{3}d_{2}C\Gamma_{1}+Krbd_{3}Cd_{2}C\Gamma_{1}+Kard_{2}+Kard_{2}G$$
  (22) $$A_{5}=-\Gamma_{1}d_{3}d_{1}brK^{2}-\Gamma_{1}d_{1}brK^{2}d_{C}-d_{C}\Gamma_{1}d_{3}brK^{2}-d_{C}\Gamma_{1}br\;K^{2}d_{C}-d_{2}raK^{2}-d_{G}raK^{2}-\Gamma_{1}d_{3}d_{1}aK^{2}-\Gamma_{1}d_{1}aK^{2}d_{2}C-d_{2}C\Gamma_{1}d_{3}aK^{2}-d_{2}C\Gamma_{1}aK^{2}d_{2}C$$

## Appendix B

The coefficients of the eigenvalues in Eq. (10) are given as follows:

(23) $$\alpha =\Gamma_{2}^{2}d_{1}\zeta -\Gamma_{2}b_{2}d_{1}^{2}\Gamma_{2}b_{2}d_{1}d_{2}-(\Gamma_{2}d_{2}(d_{1}(b_{2}+1)\zeta )+(d_{2}d_{1}b_{2}+1)\zeta )b_{2}d_{1}$$

(24) $$\beta =\pm \sqrt{(d_{2}^{2}b_{1}+1)\zeta d_{1}b_{2}+\tau b_{2}^{2}d_{1}^{2}d_{2}\zeta \Gamma_{2}\eta b_{2}^{2}d_{2}^{2})-2{\left(d_{2}d_{1}b_{2}\zeta b_{2}d_{2}^{2}-2\Gamma 2d_{2}d_{1}\zeta b_{2}^{2}d_{1}d_{2}^{2}+2\Gamma_{2}d_{2}\zeta \right)}^{2}}$$

## Appendix C

The Jacobian matrix $J_{4}={\left(a_{ij}\right)}_{4\times 4}$ is associated with the system of equations, and the roots of its characteristic polynomial determine the stability of the system. The characteristic polynomial is given by:

$$\sum_{i=0}^{4}A_{i}\lambda^{4-i}=0$$

where $A_{0}=1$, and the coefficients $A_{i}$ (i.e., $A_{1},A_{2},A_{3},A_{4}$) are derived from the elements of the Jacobian matrix. The coefficients $A_{1},A_{2},A_{3},A_{4}$ of the characteristic polynomial are functions of the Jacobian elements:

$$A_{1}=-(a_{1,1}+a_{2,2}+a_{3,3}+a_{4,4})$$

The expressions for $A_{2},A_{3},A_{4}$ involve more complex sums and products of the Jacobian elements, which are explicitly defined in terms of the matrix components. According to the Routh-Hurwitz criterion, a system is locally asymptotically stable if all the roots of the characteristic equation have negative real parts. This can be ensured if all the Hurwitz determinants are positive, leading to the following conditions:

$$A_{1}>0,\;A_{3}>0,\;A_{1}A_{2}>A_{3}$$

Additionally, the inequality:

$$A_{3}>\sqrt{A_{1}(A_{1}A_{4}-A_{2}A_{3})}$$

or

$$A_{1}A_{2}A_{3}>A_{3}^{2}+A_{1}^{2}A_{4}$$

must be satisfied to ensure the system's stability. Given that $A_{1}<0$ and $A_{3}<0$, specific conditions based on the signs of the matrix elements emerge: For instance, $a_{1,2}<0$, $a_{2,1}>0$, $a_{2,3}<0$, and so forth, indicating interactions between the system's variables, which can influence stability. The condition $A_{1}A_{2}A_{3}>A_{3}^{2}+A_{1}^{2}A_{4}$ is satisfied, indicating that the system is locally asymptotically stable. However, since $A_{1}$ and $A_{3}$ are negative, the system exhibits complex dynamics. The mention of spiralling behaviour indicates the presence of complex eigenvalues (corresponding to oscillatory behaviour). If one complex eigenvalue has a positive real part and the other has a negative real part, the equilibrium is a saddle-point focus.

The full dynamical equilibrium $E_{4}$ is unstable, with complex eigenvalues leading to spiralling trajectories. This corresponds to the system's behaviour, where upstream and downstream dynamics (e.g., bed usage in departments) impact the system. Upstream refers to the discharge or mortuary, while downstream refers to active departments, such as level-3 beds. By applying the Routh-Hurwitz criterion and analyzing the Jacobian elements, we have identified the necessary conditions for local asymptotic stability. The inequalities involving $A_{1},A_{2},A_{3},A_{4}$ confirm stability, but the presence of spiralling behaviour suggests that $E_{4}$ is unstable. The system exhibits complex oscillatory dynamics near this equilibrium point, possibly leading to saddle-point behaviour.
